# Economic Burden Associated With Cardiac Implantable Electronic Device (CIED) Infections in New South Wales, Australia: A Population‐Based Study Using Linked Administrative Data

**DOI:** 10.1002/joa3.70237

**Published:** 2025-12-02

**Authors:** Md Shajedur Rahman Shawon, Behnoosh Hosseinloui Khalaj, Michelle Hill, Gabrielle Challis, Liesl Strachan, Louisa Jorm

**Affiliations:** ^1^ Centre for Big Data Research in Health University of New South Wales Sydney Australia; ^2^ Medtronic Australasia Sydney Australia

**Keywords:** cardiac device infection, CIED infection, complete system removal, device explant, hospital costs, lead extraction, total costs

## Abstract

**Background:**

Cardiac implantable electronic device (CIED) infections are a serious complication that occurs in ~1%–3% of device recipients. However, data on total healthcare costs associated with CIED infections in Australia are scant. This study aims to comprehensively estimate the total healthcare costs of CIED infections in Australia.

**Methods:**

This retrospective cohort study included patients 18+ years diagnosed with CIED infections between July 2017 and September 2022 in New South Wales. Using linked administrative data, costs were estimated for in‐hospital care, emergency department visits, outpatient services, Medicare claims, ambulance transport, and dispensed medications in the period from 28 days before to 42 days after CIED infection‐related hospitalizations.

**Results:**

We identified 726 patients with CIED infections, of whom 233 (32.1%) died during a mean follow‐up of 35.6 months. The average treatment costs of $77 746, predominantly driven by hospital expenses (88.3%). Key hospital cost drivers included device type, mechanical ventilation, intensive care unit (ICU) stays, temporary pacing, lengths of stay, high‐risk patients, and multiple comorbidities. Patients undergoing complete system removal with reimplantation (31.7% of patients) had the highest costs ($120 792), followed by patients with complete system removal only (15.7%; $98 453), and without system removal (52.6%; $45 649). For patients undergoing complete system removal and/or reimplantation procedures, the cost varied by device type ($90 089 for pacemaker patients, $111 677 for cardiac resynchronization therapy (CRT)‐pacemaker, $128 864 for implantable cardiac defibrillators, and $148 888 for CRT‐defibrillator patients).

**Conclusions:**

Our findings highlight the substantial health care costs associated with CIED infections, with wide variations across patient factors and clinical care pathways.

## Graphical abstract

This population‐based study shows CIED infections generate high healthcare costs in Australia, averaging AUD $77 746 per patient. Costs were driven by hospitalization and varied significantly by device type, comorbidities, and treatment pathway.

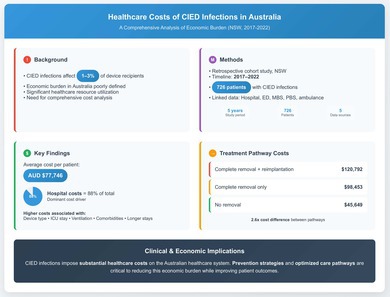

## 1 Introduction

The use of cardiac implantable electronic devices (CIEDs) is increasing worldwide [1, 2], including in Australia [3], due to an aging population and advances in cardiac care. However, the rise in CIED implantations has led to a corresponding increase in the number of patients experiencing complications [2, 4, 5]. Affecting approximately 1%–3% of patients, CIED infections pose significant clinical challenges, requiring complex and expensive treatments such as prolonged hospital stays, intensive care unit admissions, extensive diagnostics, prolonged antibiotic therapy, and often device and lead extraction followed by reimplantation [2, 4, 6, 7]. These infections not only threaten patient safety but also place a considerable economic burden on healthcare systems [8]. Understanding the true healthcare costs of CIED infections is crucial to inform decision‐making about the cost‐effectiveness of potential prevention strategies.

The current evidence on the economic burden of CIED infections is limited by significant gaps, particularly the lack of comprehensive and contemporary real‐world data on healthcare costs beyond the inpatient setting. Most existing research, primarily from the United States [9, 10, 11, 12], Canada [13], and Europe [14, 15], focuses on hospital‐related resource utilization and costs, overlooking critical aspects such as prehospital care, postdischarge services, and medications. In Australia, data are particularly sparse, with only one study [16], showing a large discrepancy in costs per hospital admission between the Barwon Health region ($98 097) and the state of Victoria ($19 403), highlighting inconsistencies and the need for more reliable data. There is also limited evidence describing the full spectrum of treatment pathways, including cases with no system removal, complete removal without reimplantation, and complete removal followed by reimplantation. The complexity of the Australian healthcare system, which includes a mix of public and private sectors with varying funding arrangements for patients with and without private health insurance, further complicates cost estimation. Therefore, understanding costs considering hospital funding types and private health insurance involved in treatment pathways is essential to provide a clearer picture of the true economic impact of CIED infections in Australia.

The aim of this study was to provide a comprehensive assessment of the economic burden of CIED infections in Australia by evaluating healthcare service utilization and associated costs occurring before, during, and after hospitalization, including expenses related to in‐hospital care, emergency departments, outpatient services, Medicare‐billed subsidies, ambulance services, and dispensed medications.

## 2 Materials and Methods

### 2.1 Study Setting

This was a population‐based cohort study in New South Wales (NSW), Australia's most populous state, with approximately 8 million residents, and covering a geographical area of more than 800,000 km^2^. The majority of the NSW population (75%) lives in major cities, while the remainder lives in areas classified as regional or remote [17]. The Australian healthcare system operates under a complex funding arrangement that integrates both public and private sectors [18]. Public hospitals are managed by state governments and are jointly funded by state and Commonwealth governments. This arrangement allows public hospitals to offer treatment to public patients at no cost to the patient, with no claims made against the national publicly funded insurance scheme, Medicare. In contrast, services provided in private hospitals operate under a different funding model, primarily supported by private health insurance, with benefits paid to clinicians through Medicare Benefits Schedule (MBS) claims, and patient out‐of‐pocket payments paid both to clinicians and hospitals. Patients in Australia have the option to choose between public or private care when hospitalized, influencing how their care is funded and billed. This mixed model allows for broad access to healthcare services, but it also creates a complex landscape of funding and billing that varies significantly depending on the patient's choice of public or private care [18]. Out‐of‐pocket payments have not been considered in this study.

### 2.2 CIED Infection Cohort

Patients with CIED infections were identified using the NSW Admitted Patient Data Collection (APDC), which records demographic characteristics, diagnoses, and procedures for all hospital separations from public and private hospitals in NSW. Diagnoses are coded according to the International Statistical Classification of Diseases and Related Health Problems, Tenth Revision, Australian Modification (ICD‐10‐AM) [19], and procedures are coded according to the Australian Classification of Health Interventions (ACHI) [20]. From 1 July 2017 to 30 September 2022 (public hospitals) or 30 June 2021 (private hospitals, the most recent data available), we identified CIED infections using the ICD‐10‐AM code, T82.71 (Infection and inflammatory reaction due to electronic cardiac device) in any APDC diagnosis field. Multiple consecutive hospital episodes for the same patient, including same‐day discharges and transfers followed by another acute admission within 24 h, were combined into a single hospital stay. We excluded episodes with potential linkage errors, such as discharges before admissions or admissions after death dates.

### 2.3 Linked Datasets to Identify Resource Utilization and Cost Estimates

**FIGURE 1:**
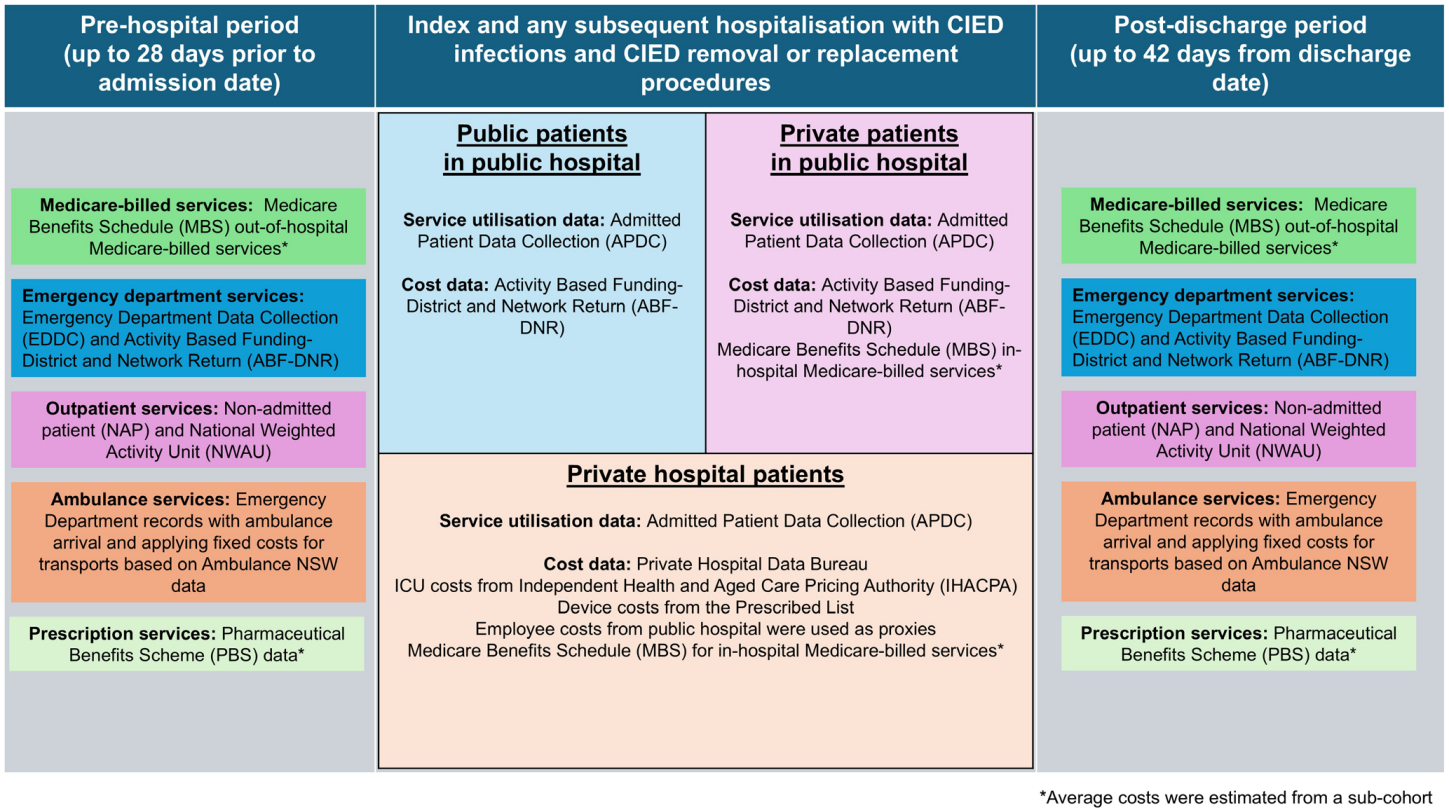
Overview of datasets detailing service utilization and cost data for CIED infection patients.

**FIGURE 2:**
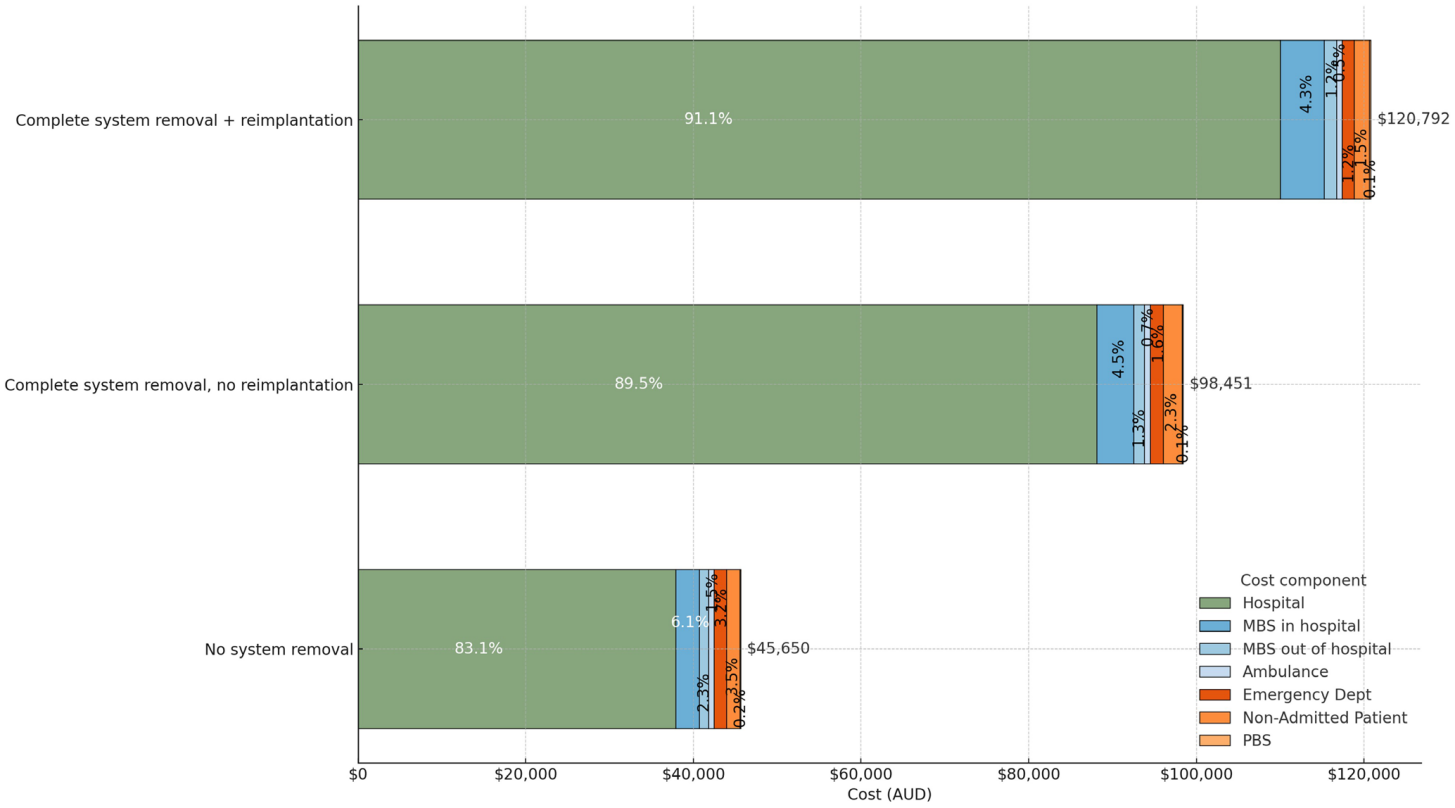
Cost breakdown of treatment pathways following CIED infection.

A comprehensive range of linked person‐level datasets was used to capture healthcare service utilization and associated costs for patients hospitalized with CIED infections (Figure 1), provides an overview of these datasets (Figure 2).

#### 2.3.1 In‐Hospital Services and Costs

All hospital admissions for CIED infections were identified, including the index admission and any subsequent admissions where a CIED infection diagnosis was recorded, using APDC data from the date of the first CIED infection admission through to the end of the study period (30 September 2022 for public hospitals and 30 June 2021 for private hospitals), capturing associated in‐hospital services and costs. Treatment pathways were categorized into three groups: no system removal, complete system removal without reimplantation, and complete system removal with reimplantation. These categories were defined using relevant ACHI codes [20] from the principal or additional procedure fields during the initial or subsequent hospitalizations (Table S1). Other key variables included device type, patient demographics, length of stay, use of mechanical ventilation, admission to intensive care unit (ICU), hospital funding type, and death status. Comorbidities were identified from primary and additional diagnosis fields recorded during the initial CIED infection hospitalization or any hospitalizations up to 24 months prior. High‐risk patients were defined based on the criteria from the Worldwide Randomized Antibiotic Envelope Infection Prevention Trial (WRAP‐IT), a multicenter, randomized, controlled, prospective interventional clinical trial [21], including those undergoing CIED generator reimplantation, system upgrades with or without new leads, CIED pocket or lead revision, or an initial cardiac resynchronization therapy (CRT) procedure (ACHI codes in Table S2).

Hospital costs were calculated using Activity Based Funding‐District and Network Return (ABF‐DNR) data for public hospital admissions, which is a mandatory submission to the NSW Ministry of Health containing patient activity, utilization data, and general ledger expenses to calculate hospital costs [22]. These data are audited locally and inform both state and national pricing and data submissions. We estimated private hospital costs based on procedure and device types, with accommodation and theater costs sourced from the Private Hospital Data Bureau [23], ICU costs from the Independent Health and Aged Care Pricing Authority (IHACPA) [24], and device costs from the Prescribed List [25]. Due to data limitations, public hospital employee costs were used as proxies for private hospital employee costs.

#### 2.3.2 Out‐of‐Hospital Services and Costs

The time period for calculating costs for out‐of‐hospital services in this analysis was from 28 days prior to the index CIED‐related admission to 42 days after discharge from CIED‐related hospital stays. This window was selected based on the distribution of out‐of‐hospital costs relative to the index admission dates for CIED infections, as well as expert advice from clinicians. Emergency department (ED) visits were identified using the NSW Emergency Department Data Collection (EDDC), and associated costs were derived from ED data merged with ABF‐DNR data. Nonadmitted patient (NAP) care was assessed using NAP data, limited to the same timeframe, with costs estimated from Non‐Admitted Patient National Weighted Activity Unit (NWAU) data [22]. Ambulance costs were estimated from ED records showing ambulance as the mode of arrival, applying fixed costs for emergency and nonemergency transport based on Ambulance NSW data [26].

#### 2.3.3 Medicare‐Billed Services and Costs

Linked Medicare data (Medicare Benefits Schedule [MBS] and Pharmaceutical Benefits Scheme [PBS]) were only available for a subset of the cohort hospitalized in the period July 2017 to December 2018. For these patients, we identified both in‐hospital and out‐of‐hospital Medicare‐billed services in the period 28 days before admission up to 42 days after discharge from CIED infection‐related hospital stays. Costs were calculated as “benefits paid” (75% of the schedule fee) plus 25% of the schedule fee, excluding out‐of‐pocket costs. Costs for dispensed antibiotics relevant to CIED infections (Table S3) within the specified timeframe were estimated using linked PBS data.

#### 2.3.4 Mortality

Mortality data were obtained from the NSW Registry of Births, Deaths, and Marriages (RBDM).

### 2.4 Ethics Statement

Ethics approval for the study was provided by the NSW Population and Health Services Research Ethics Committee (Ref: 2019/ETH00436). The NSW Centre for Health Record Linkage (https://www.cherel.org.au/) performed the data linking through a probabilistic method, with an estimated false positive rate of 0.5% (5 per 1000) [27].

### 2.5 Statistical Analysis

Descriptive statistics, including frequencies and percentages for categorical variables and means with standard deviations (SD) for continuous variables, were used to describe patient and clinical characteristics. Healthcare utilization data were presented as frequencies and percentages. For the costing analysis, we assessed total mean costs, including in‐hospital costs, MBS‐billed costs (in and out of hospital), ambulance, ED visit, outpatient, and PBS costs for all patients. To estimate MBS and PBS costs for the entire study period, we assigned average costs from the subset for whom linked data were available (*n* = 220). Average in‐hospital MBS costs were calculated based on hospital type, private health insurance status, and were indexed according to the relevant financial year. All patients were assigned the same average out‐of‐hospital MBS costs, also indexed according to financial year. All patients were assigned the same average cost for dispensed antibiotics relevant to CIED infections, without indexation adjustments. Cost estimates per patient were calculated separately according to demographic, patient, and clinical factors.

All analyses were conducted using SAS version 9.4. All costs are presented in Australian dollars (AUD) based on the year of expenditure.

## 3 Results

### 3.1 Characteristics of Patients With CIED Infections

Table 1 outlines the key characteristics of the 726 patients identified with CIED infections during the study period. More than half (54.3%) of the patients were aged 75 years or older, and nearly two‐thirds (68.2%) were male. A significant proportion (81.4%) of patients were not classified as high risk, while 18.6% were considered high risk. The majority were treated in public hospitals only (63.9%), followed by those with both public and private hospital episodes (23.7%), and those treated in private hospitals only (12.4%).

**TABLE 1:** Characteristics of patients with CIED infections in 2017–2022 in NSW.

	Number of patients	Column %
Age group		
Under 55	105	14.5
55–75	227	31.3
75 plus	394	54.3
Sex		
Male	495	68.2
Female	231	31.8
Device type		
CRT‐D	45	6.2
CRT‐P	38	5.2
ICD	100	13.8
PPM	197	27.1
Not identified as no procedure was performed	346	47.7
Risk group		
Not a high‐risk patient	591	81.4
High‐risk patient	135	18.6
Comorbidities		
Atrial fibrillation	269	37.1
Cardiomyopathy	102	14
Stroke	60	8.3
Myocardial infarction	82	11.3
Obesity	148	20.4
Chronic kidney disease	221	30.4
Diabetes	237	32.6
Chronic obstructive pulmonary disease	134	18.5
Hypertension	522	71.9
Peripheral vascular disease	202	27.8
Coronary artery disease	390	53.7
Congestive heart failure	355	48.9
Index Year		
2017	80	11
2018	159	21.9
2019	154	21.2
2020	139	19.1
2021 (private hospitals up to and including June only)	118	16.3
2022 (public hospitals only)	76	10.5
Hospital type		
Public hospital only	464	63.9
Private hospital only	90	12.4
Mix of public and private episodes	172	23.7
Number of hospital stays during the study period*		
One stay	565	77.8
Two stays	127	17.5
Three or more stays	34	4.7
Mechanical ventilation		
No mechanical ventilation	674	92.8
At least 1 h of mechanical ventilation	52	7.2
Intensive Care Unit (ICU)		
No ICU	476	65.6
Up to 24 h in ICU	74	10.2
More than 24 h in ICU	176	24.2
Insertion of temporary CIED (pacing)		
No	646	89
Yes	80	11
Total length of stay		
7 days and under	142	19.6
8–4 days	86	11.8
15–21 days	79	10.9
22 days and over	419	57.7
Pathway		
Complete system removal, no reimplantation	114	15.7
Complete system removal, subsequent device implanted	230	31.7
No complete system removal	382	52.6
Vital status at the end of the study period		
Alive	493	67.9
Dead	233	32.1
**All**	**726**	**100**

Abbreviations: CRT‐D, cardiac resynchronization therapy‐defibrillator; CRT‐*P*, cardiac resynchronization therapy‐pacemaker; ICD, implantable cardioverter defibrillator; PPM, permanent pacemaker.

* We considered episodes involving transfers between hospitals or changes in care types (e.g., from acute to subacute) as a continuation of the same hospital stay.

During a median follow‐up time of 35.6 months (IQR 19.6, 49.8) from the first admission with CIED infection, 52.6% of patients did not undergo complete system removal, 15.7% had complete system removal without reimplantation, and 31.7% had complete system removal followed by subsequent implantation of a new device. Of patients with a CIED‐related procedure (i.e., extraction and/or reimplantation of a device), the majority had a pacemaker (PM) procedure (51.8%), 10% had a CRT‐P procedure, 26.3% had an implantable cardioverter‐defibrillator (ICD) procedure, and 11.8% had a CRT‐D procedure. Among patients hospitalized with CIED infections, 77.8% had one hospital stay, 17.5% had two stays, and 4.7% had three or more stays, resulting in an average of 1.36 stays per patient. During the follow‐up period, 32.1% of the overall cohort died.

### 3.2 Healthcare Costs Across Different Services

Table 2 shows the average costs across different services, according to patient pathways and characteristics. Among the 726 patients with CIED infections, the mean cost for hospital services was $68 651. Patients who had mechanical ventilation had much higher hospital costs ($197 001) than those without ventilation ($58 749). Patients who had ICU stays for 24 h or more had higher costs ($136 442) compared to those with no ICU use ($42 296). Patients who had complete system removal with subsequent implantation had higher hospital costs ($110 018) compared to those without complete system removal ($37 935). High‐risk patients incurred higher hospital costs ($112 908 vs. $58 542 for non–high‐risk patients). Hospital costs were higher for patients with longer stays ($98 790 for stays over 22 days vs. $15 626 for stays of 7 days or less). Comorbidities were also associated with increased hospital costs, and deceased patients had higher hospital costs than those alive at the end of the study period ($84 077 vs. $61 361). Hospital costs were higher for males than females ($71 649 vs. $62 228) and for those under 55 compared to those aged 75 and older ($91 225 vs. $59 497). Hospital costs varied between patients admitted to public hospitals only ($59 571), private hospitals only ($72 720), and both public and private hospitals ($91 019).

**TABLE 2:** Estimated costs per patient for in‐hospital and out‐of‐hospital healthcare services and total cost according to patient and care‐related factors.

Factor	Hospital	MBS^a^ in hospital	MBS^a^ out of hospital	Ambulance	Emergency department	Nonadmitted patient	PBS^b^	Total
Demographics								
Age group								
Under 55	$91 225	$2961	$1401	$476	$1327	$2407	$125	$99 923
55–75	$74 098	$3269	$1212	$611	$1527	$1871	$109	$82 698
75 plus	$59 497	$4396	$1186	$799	$1440	$1559	$106	$68 983
Sex								
Male	$71 649	$3837	$1230	$686	$1401	$1954	$110	$80 868
Female	$62 228	$3835	$1215	$709	$1557	$1404	$109	$71 057
Clinical characteristics								
Device type								
CRT‐D	$138 388	$4252	$1553	$610	$1821	$2125	$140	$148 888
CRT‐P	$94 265	$11 739	$2030	$572	$1318	$1571	$183	$111 677
ICD	$119 705	$2808	$1261	$719	$1554	$2704	$113	$128 864
PPM	$80 659	$4620	$1253	$651	$1269	$1525	$112	$90 089
No procedure identified	$35 176	$2765	$1068	$735	$1491	$1635	$95	$42 966
Risk group								
Not high risk	$58 542	$3767	$1212	$685	$1442	$1768	$108	$67 524
High risk	$112 908	$4138	$1284	$730	$1489	$1830	$115	$122 494
Comorbidities								
Atrial fibrillation	$82 716	$3965	$1208	$732	$1452	$2177	$108	$92 359
Cardiomyopathy	$100 113	$2959	$1200	$585	$1629	$2472	$108	$109 064
Stroke	$97 114	$2600	$1130	$752	$1312	$1497	$101	$104 506
Myocardial infarction	$76 800	$2794	$1035	$860	$1475	$1831	$93	$84 888
Obesity	$93 568	$4011	$1311	$770	$1607	$1975	$117	$103 359
Chronic kidney disease	$92 917	$3560	$1196	$897	$1574	$2213	$107	$102 465
Diabetes	$78 109	$3238	$1152	$844	$1554	$1752	$103	$86 751
COPD	$88 595	$2649	$1122	$948	$1739	$2097	$101	$97 251
Hypertension	$69 365	$4090	$1242	$776	$1513	$1791	$111	$78 889
Peripheral vascular disease	$85 820	$4074	$1293	$895	$1597	$2280	$116	$96 075
Coronary artery disease	$72 917	$3940	$1192	$756	$1534	$1828	$107	$82 275
Congestive heart failure	$85 114	$3497	$1203	$788	$1516	$2145	$108	$94 371
Care and hospital factors								
Index year								
2017	$70 978	$4142	$1264	$721	$1360	$1953	$115	$80 532
2018	$77 390	$3547	$1267	$854	$1779	$2047	$115	$87 000
2019	$69 811	$4147	$1205	$798	$1487	$1967	$109	$79 524
2020	$73 627	$5998	$1440	$569	$1378	$1705	$128	$84 844
2021	$61 265	$2491	$1027	$714	$1490	$1655	$90	$68 733
2022	$47 938	$1624	$1051	$316	$861	$983	$91	$52 864
Hospital type								
Public only	$59 571	$1517	$1104	$788	$1645	$2027	$99	$66 750
Private only	$72 720	$9646	$1351	$203	$455	$407	$121	$84 904
Mixed	$91 019	$7053	$1488	$695	$1448	$1829	$134	$103 665
Number of hospital stays								
One stay	$62 671	$2506	$903	$617	$1308	$1379	$81	$69 464
Two stays	$79 332	$5765	$1802	$870	$1724	$3062	$162	$92 716
Three or more stays	$128 135	$18 742	$4430	$1311	$2811	$3632	$397	$159 459
Mechanical ventilation								
No	$58 749	$3745	$1211	$691	$1449	$1727	$108	$67 681
At least 1 h	$197 001	$5011	$1411	$725	$1476	$2452	$126	$208 203
ICU								
No ICU	$42 296	$3271	$1171	$670	$1413	$1689	$105	$50 614
Up to 24 h	$76 946	$4352	$1274	$861	$1617	$1685	$115	$86 848
More than 24 h	$136 442	$5149	$1352	$688	$1485	$2063	$121	$147 300
Temporary CIED pacing								
No	$62 425	$3601	$1203	$665	$1408	$1797	$108	$71 206
Yes	$118 926	$5738	$1403	$929	$1797	$1636	$126	$130 554
Length of stay								
≤ 7 days	$15 626	$2907	$945	$476	$1313	$929	$85	$22 282
8–14 days	$26 059	$2885	$1112	$578	$1257	$1655	$100	$33 644
15–21 days	$50 479	$3239	$1187	$788	$1250	$1451	$106	$58 500
22 days and over	$98 790	$4459	$1351	$773	$1575	$2155	$121	$109 224
Treatment pathway								
Complete removal, no reimplantation	$88 119	$4427	$1250	$726	$1551	$2266	$112	$98 453
Complete removal, subsequent implant	$110 018	$5254	$1480	$660	$1416	$1831	$133	$120 792
No removal	$37 935	$2806	$1065	$704	$1442	$1603	$95	$45 649
Vital status								
Alive	$61 361	$4019	$1259	$543	$1347	$1662	$112	$70 303
Dead	$84 077	$3450	$1154	$1012	$1670	$2027	$104	$93 495
All	$68 651	$3836	$1225	$694	$1451	$1779	$110	$77 746

Abbreviations: CRT‐D, cardiac resynchronization therapy‐defibrillator; CRT‐*P* , cardiac resynchronization therapy‐pacemaker; ICD, implantable cardioverter defibrillator; PPM, permanent pacemaker.

^a^ MBS = Medicare Benefits Schedule, private patients at public hospitals and all patients at private hospitals are eligible for MBS. Public patients at public hospitals are not eligible for MBS.*

^b^ PBS = Pharmaceutical Benefits Scheme.

For the period from 28 days before admission to 42 days after discharge from CIED infection‐related hospitalizations, the estimated mean costs were $1225 for out‐of‐hospital MBS services, $694 for ambulance, $1451 for ED, $1779 for NAP, and $110 for medication costs. Compared to those without complete system removal, patients with complete system removal and subsequent device implantation had higher in‐hospital ($5254 vs. $2806) and out‐of‐hospital MBS costs ($1480 vs. $1065). Those with temporary pacing also had higher in‐hospital ($5738 vs. $3601) and out‐of‐hospital MBS costs ($1403 vs. $1203) than those without. Additionally, patients requiring mechanical ventilation had higher MBS costs both in‐hospital ($5011 vs. $3745) and out‐of‐hospital ($1411 vs. $1211). Ambulance costs were substantially higher in deceased patients ($1012 vs. $543 for survivors), while ED costs were also higher in deceased ($1670 vs. $1347) and temporary pacing patients ($1797 vs. $1408). NAP costs were higher among patients with mechanical ventilation, cardiomyopathy, those under 55, and those with longer lengths of stay. Medication costs were relatively consistent across various factors.

### 3.3 Total Costs Associated With Treating CIED Infections

Table 2 shows the average total costs associated with treating CIED patients, according to patient pathways and characteristics. The average total cost per patient was $77 746. Patients with complete system removal and subsequent implantation had the highest costs ($120 792), while those without complete system removal had lower costs ($45 649) (Figure 2). For patients undergoing complete system removal and/or reimplantation procedures, the cost varied by device type ($90 089 for PM patients, $111 677 for CRT‐P patients, $128 864 for ICD patients, and $148 888 for cardiac CRT‐D patients). High risk of infection patients incurred significantly higher costs ($122 494) compared to those not at high risk ($67 524). Costs were higher for patients who died during the study period ($93 495 vs. $70 303). Mechanical ventilation was a major cost driver, with total costs reaching $208 203 per patient on average. ICU stays over 24 h also had high costs ($147 300). Temporary pacing increased costs notably ($130 554 vs. $71 206 without pacing). Length of stay over 22 days resulted in higher costs ($109 224). Patients with comorbidities such as cardiomyopathy ($109 064), stroke ($104 506), and chronic obstructive pulmonary disease ($97 251) incurred higher costs than those without these comorbidities. Patients who were admitted to both public and private hospitals had the highest total average cost ($103 665), followed by those admitted to private hospitals only ($84 904) and those admitted to public hospitals only ($66 750).

## 4 Discussion

In this contemporary cohort of CIED patients with infection, the average cost of in‐hospital and out‐of‐hospital treatment was $77 746. Hospital expenses were the major contributor to the overall cost of treating CIED infections (88.3% of total costs), with significant cost drivers including high risk of infection status (CRT‐D, revision and reimplantation procedures), mechanical ventilation, ICU stays exceeding 24 h, complete system removal with reimplantation, ICD and CRT‐D device types, and temporary pacing. Factors such as longer length of stay and the presence of specific comorbidities, including cardiomyopathy and other chronic conditions, further increased hospital costs.

In our previous study [7], among the 37 675 patients who underwent CIED procedures in NSW (2016–21), 1.1% were hospitalized with related infections within 12 months, increasing to 1.3% within a median follow‐up of 27 months. In our current study, we examined the economic burden of CIED infections before, during, and after hospitalization, using a wide range of linked person‐level datasets from NSW to capture healthcare utilization and associated costs for patients hospitalized with CIED infections. This approach offers a more comprehensive examination of costs associated with infection than the previous Australian study [16], which reported widely varying costs—from $19 403 per hospitalization in Victoria to $98 097 per hospitalization in the Barwon Health region. The Victorian data in that study included other cardiac and vascular infections, broadening the cost range and likely including infections out of the scope of CIED infection analyses, while Barwon Health's smaller sample size may have led to inflated per‐patient costs due to limited generalizability. In contrast, our use of large datasets and specific diagnostic codes for CIED infections is more likely to yield a more accurate average cost estimate of $77 746, better reflecting the true economic burden of these infections among Australian patients. While the previous study [16] focused only on direct hospital costs, our analysis looked at costs associated with the entire patient journey, including outpatient care, ED visits, multiple admissions per patient, and prescription costs. We also provided detailed cost estimates based on patient factors such as age, sex, and comorbidities, as well as treatment factors such as device type, length of stay, ICU admission, and mechanical ventilation. This comprehensive analysis is crucial not only for evaluating the cost‐effectiveness of CIED infection prevention strategies by identifying relevant patient populations and reporting complete costs, but also for informing health system and hospital planning and guiding future treatment and practice guidelines.

In our study, hospital costs accounted for 88.3% of the total treatment costs, which aligns with findings from Canadian [13], European, and U.S. studies [9, 10, 11, 12]. For example, a study in Alberta, Canada, reported that hospitalizations accounted for around 70% of the total costs associated with CIED infections [13]. Additionally, in the U.S. Medicare population, hospitalization was the largest cost driver, with infection‐related payments contributing to more than 50% of expenditures [9]. However, comparing costs across different studies is challenging due to several factors. First, the proportion of de novo versus reimplantation procedures varies significantly, as observed in both Canadian [13] and French [14] Studies. Second, the severity of infections within the cohort, with some studies reporting major infections requiring complex interventions, affects cost variations. For instance, the U.S. Medicare study identified that device reimplantation without extraction incurred lower costs compared to cases requiring both extraction and reimplantation [11]. Finally, healthcare systems differ, with U.S. healthcare costs typically being higher due to different reimbursement structures, compared to the Australian, Canadian, and French systems.

In our study, treatment intensity was a key factor driving costs, with the highest expenses observed in patients undergoing both complete system removal and reimplantation ($120 792), followed by patients undergoing only complete system removal ($98 453), and those without complete system removal ($45 649). This cost gradient mirrors findings from other studies [9, 11, 28] that demonstrate increased costs with more complex treatment pathways. For example, the WRAP‐IT trial showed that costs varied according to treatment intensity: 16 592 USD (25 718 AUD; conversion rate 1 USD = 1.55 AUD) for infections treated without extraction, 45 694 USD (70 826 AUD) for extraction without reimplantation, and 67 586 USD (104 758 AUD) for extraction with reimplantation [28]. Similarly, the U.S. Medicare study reported costs ranging from 22 856 USD (35 427 AUD) for patients who did not undergo CIED‐related procedures to 77 397 USD (119 965 AUD) for those hospitalized without device removal [11]. A health insurer analysis from the United States also found that infection management costs ranged from 16 651 USD (25 809 AUD) for less intensive treatments to 279 744 USD (433 603 AUD) for severe sepsis cases requiring implant removal [12]. It is important to note that a large proportion of patients with a CIED infection did not have complete system removal (52.6%) despite device removal being recommended in clinical guidelines [29]. It is also interesting to note that patients in the public sector had higher rates of patients without complete system removal (64.9%) than those in the private sector (46.7%).

We found that costs were higher for males compared with females, and for younger compared with older patients. A likely explanation for these differences is variation in treatment pathways. In related work, we found that females and older patients were less likely to undergo lead extraction following CIED infection, a procedure associated with substantial costs [30]. These differences in management may therefore contribute to the observed variation in overall costs by sex and age. Among those with complete system removal, the type of device is also a key factor driving costs, with the overall cost of infection for patients who had a pacemaker procedure being $90 089, and this cost increased to $148 888 for patients with a CRT‐D procedure. As 51.8% of patients who had a device‐related procedure were pacemaker patients and only 11.8% were CRT‐D patients, this would significantly influence the overall average cost of device‐related infections. These findings consistently highlight that more complex treatment pathways, particularly those involving reimplantation, significantly increase the financial burden. However, it is important to note that these comparisons are unadjusted descriptive estimates. Patients receiving more complex devices such as CRT‐D are likely to have higher baseline comorbidity and clinical complexity, which may also contribute to higher costs. Therefore, while device type is a major driver of variation, part of the observed cost differences may reflect underlying patient characteristics rather than device complexity alone.

This is the first Australian study to examine and compare the costs of CIED infections across hospital sectors. We found that the average cost was lowest among patients treated exclusively in public hospitals ($66 750), higher for those treated only in private hospitals ($84 904), and highest for patients who received care across both sectors ($103 665). The higher costs among mixed‐sector patients likely reflect greater complexity, care transfers, and more resource‐intensive treatment pathways. However, the interpretation of these results requires caution. Public hospital costs were derived from the NSW ABF‐DNR patient‐level dataset, while private hospital costs had to be estimated using composite methods due to the absence of Australian Refine—Diagnosis‐Related Group (AR‐DRG) or patient‐level data, and these estimates were only available up to June 2021. This heterogeneity in costing methods introduces potential bias, as private hospital costs may be under‐ or overestimated depending on billing practices, negotiated charges, and case mix. Furthermore, patients who moved between public and private hospitals are likely to represent a selected group with more severe or complex disease, meaning their higher costs are not directly comparable with single‐sector patients. Despite these limitations, our analysis highlights the substantial financial burden across all hospital sectors and reinforces the importance of addressing both public and private care pathways in future strategies to prevent and manage CIED infections.

While our study did not estimate incremental costs between infection and noninfection groups, previous research [11, 13] has consistently shown substantial differences. For instance, the Alberta cohort study found an incremental cost difference of 90 620 CAD (103 303 AUD; 1 CAD = 1.14 AUD), with mean costs of 145 312 CAD (165 656 AUD) for infection patients versus 34 264 CAD (39 066 AUD) for noninfection patients [13]. Similarly, in the U.S. Medicare study, patients with CIED infections incurred medical costs 2.4 times higher than those without infections, with an incremental difference of 57 322 [11]. These findings demonstrate the significant financial impact of CIED infections, particularly driven by hospitalizations and intensive treatments, consistently leading to higher overall healthcare expenditures. Patients who died (almost one‐third of all patients) had higher costs than those who did not ($93 495 versus $70 303), reflecting greater use of hospital resources, including ICU stays, in these patients.

A key strength of this study is that it is the first Australian investigation to comprehensively examine CIED infection costs along the treatment pathway and by various patient factors. We estimated expenses for in‐hospital care (including multiple admissions), emergency department visits, outpatient services, Medicare claims, ambulance transport, and medications, covering a 28 day pre‐ to 42 day post‐hospitalization period. The population‐based nature of our datasets ensures broad generalizability, and we reported costs across public and private hospitals. Additionally, detailed cost estimates according to various patient factors, clinical care management, and treatment pathways, offering a more comprehensive understanding of the economic burden, were provided.

A key limitation of our study is the use of a single diagnostic code (ICD‐10‐AM T82.71) to identify CIED infections, which could encompass a wide range of infection severities, from localized infections to severe conditions such as bacteremia and septic shock [8]. There are no validation studies comparing ICD codes with infection surveillance data in Australia. However, a Canadian study reported that the use of code T82.7 achieved an area under the curve of 0.936 when benchmarked against surveillance data [31]. A reliance on administrative claims data meant that we lacked detailed clinical information on patient symptoms and clinical course. Additionally, the use of administrative data restricted our ability to identify the specific type of device implanted prior to 1 July 2017, unless a subsequent procedure, such as a revision or removal, was performed. It is also worth noting that patients undergoing a reimplantation procedure during the follow‐up period were likely to receive the same device type, as device upgrades are generally uncommon. An important limitation is that MBS and PBS data were only available for a subset of patients (*n* = 220 for MBS; *n* = 137 for PBS) from 2017–2018, and we could not compare their demographic or clinical characteristics with the broader cohort. Average costs from this subgroup were extrapolated across the full cohort, adjusted for insurance type, hospital type, and year of admission. Although this extrapolation may have introduced some uncertainty, the contribution of MBS and PBS to overall costs was relatively small compared with hospitalization costs, which were available for all patients. Therefore, our total cost estimates are unlikely to be materially affected by potential differences in representativeness of the MBS/PBS subgroup. As discussed earlier, differences in costing methods between public and private hospitals (ABF‐DNR versus composite estimates) introduce heterogeneity that may bias sector‐level comparisons. In particular, private hospital costs were based on less granular data, which could result in under‐ or overestimation relative to public hospital costs. Furthermore, the public hospital cost data were drawn from NSW's ABF‐DNR system, limiting the direct applicability of our findings to other healthcare systems. Since the database was primarily designed for hospital financing rather than epidemiological research, the accuracy of the data depends on hospital coding practices, which could affect the reliability of the results.

## 5 Conclusions

In conclusion, the average total cost per patient for treating CIED infections was $77 746. Hospital expenses accounted for 88.3% of the total costs, with significant cost drivers including high risk of infection status (CRT‐D or revision or reimplantation procedures), mechanical ventilation, ICU stays exceeding 24 h, complete system removal with reimplantation, device type, and temporary pacing. These findings highlight the substantial economic burden of CIED infections in Australian patients.

## Funding

This work was supported by the National Health and Medical Research Council (2013323) and Medtronic Australasia Pty Ltd.

## Conflicts of Interest

This work was supported by Medtronic Australasia Pty Ltd. through funding and awarded to the Centre for Big Data Research in Health, University of New South Wales. Authors from Medtronic Australasia (MH, LS, and GC) contributed to the study conception and design, interpreting data, critically reviewing the manuscript, and approving the manuscript. They had no access to the raw study data and did not participate in the statistical analysis. Authors from the Centre for Big Data Research in Health, University of New South Wales (BH and MS) performed the statistical analysis. Authors from the Centre for Big Data Research in Health, University of New South Wales (MS, BK, and LJ) contributed to the study conception and design, interpreting data, drafting, critically reviewing, and approving the manuscript, and had the final responsibility for the decision to submit the paper for publication. The dataset includes devices manufactured by Medtronic and any other company that supplies such devices in Australia and New Zealand.

## Supporting information


**Table S1:** ACHI codes to define CIED removal and replacement procedures.
**Table S2:**. ACHI codes to define high‐risk CIED patients.
**Table S3:**. Antibiotic medicines.

## Data Availability

Due to legal and ethical restrictions, the de‐identified linked administrative health data used in this research cannot be publicly shared.
