# Supplementary material for: Economic Burden Associated With Cardiac Implantable Electronic Device (CIED) Infections in New South Wales, Australia: A Population‐Based Study Using Linked Administrative Data
Source: J Arrhythm. 2025 Dec 2;41(6):e70237. doi: 10.1002/joa3.70237 (PMC12670187; doi:10.1002/joa3.70237)
Supplement: Supplementary file 1 — Table S1: ACHI codes to define CIED removal and replacement procedures. Table S2:. ACHI codes to define high‐risk CIED patients. Table S3:. Antibiotic medicines. [file JOA3-41-e70237-s001.docx]

**Economic burden associated with cardiac implantable electronic device (CIED) infections in New South Wales, Australia: a population-based study using linked administrative data**

**Supplementary materials**

**Table S1: ACHI codes to define CIED removal and replacement procedures**

| Code | Description | Category |
| --- | --- | --- |
| 38368-04 | Removal of permanent transvenous electrode of left ventricle for cardiac defibrillator | Removal |
| 38654-02 | Removal of permanent left ventricular electrode for cardiac pacemaker via thoracotomy or sternotomy | Removal |
| 38456-34 | Removal of permanent epicardial electrode for cardiac defibrillator via thoracotomy or sternotomy | Removal |
| 38456-33 | Removal of permanent epicardial electrode for cardiac defibrillator via subxyphoid approach | Removal |
| 38456-27 | Removal of permanent epicardial electrode for cardiac pacemaker via thoracotomy or sternotomy | Removal |
| 38456-26 | Removal of permanent epicardial electrode for cardiac pacemaker via subxyphoid approach | Removal |
| 38654-05 | Removal of permanent left ventricular electrode for cardiac defibrillator via thoracotomy or sternotomy | Removal |
| 38368-02 | Removal of permanent transvenous electrode of left ventricle for cardiac pacemaker | Removal |
| 38358-03 | Removal of permanent transvenous electrode of other heart chamber(s) for cardiac defibrillator using extraction device | Removal |
| 90203-07 | Removal of cardiac defibrillator generator | Removal |
| 38358-01 | Removal of permanent transvenous electrode of left ventricle for cardiac pacemaker using extraction device | Removal |
| 38358-00 | Removal of permanent transvenous electrode of other heart chamber(s) for cardiac pacemaker using extraction device | Removal |
| 38350-02 | Removal of permanent transvenous electrode of other heart chamber(s) for cardiac pacemaker | Removal |
| 38353-02 | Removal of cardiac pacemaker generator | Removal |
| 38358-02 | Removal of permanent transvenous electrode of left ventricle for cardiac defibrillator using extraction device | Removal |
| 38350-04 | Removal of permanent transvenous electrode of other heart chamber(s) for cardiac defibrillator | Removal |
| 38368-01 | Replacement of permanent transvenous electrode of left ventricle for cardiac pacemaker | Replacement |
| 38654-04 | Replacement if permanent left ventricular electrode for cardiac defibrillator via thoracotomy of sternotomy | Replacement |
| 38654-01 | Replacement of permanent left ventricular electrode for cardiac pacemaker via thoracotomy or sternotomy | Replacement |
| 38350-03 | Replacement of transvenous electrode of other heart chamber(s) for cardiac defibrillator | Replacement |
| 38353-01 | Replacement of cardiac pacemaker generator | Replacement |
| 38456-30 | Replacement of permanent epicardial electrode for cardiac defibrillator via subxiphoid approach | Replacement |
| 38456-24 | Replacement of permanent epicardial electrode for cardiac pacemaker via thoracotomy or sternotomy | Replacement |
| 38456-23 | Replacement of permanent epicardial electrode for cardiac pacemaker via subxiphoid approach | Replacement |
| 38393-01 | Replacement of cardiac defibrillator generator | Replacement |
| 38368-03 | Replacement of transvenous electrode of left ventricle for cardiac defibrillator | Replacement |
| 38456-31 | Replacement of permanent epicardial electrode for cardiac defibrillator via thoracotomy or sternotomy | Replacement |
| 38350-01 | Replacement of permanent transvenous electrode of other heart chamber(s) for cardiac pacemaker | Replacement |

**Table S2: ACHI codes to define high-risk CIED patients**

| Code | Description |
| --- | --- |
| 38350-01 | Replacement of permanent transvenous electrode of other heart chamber(s) for cardiac pacemaker |
| 38350-03 | Replacement of transvenous electrode of other heart chamber(s) for cardiac defibrillator |
| 38353-01 | Replacement of cardiac pacemaker generator |
| 38368-00 | Insertion of permanent transvenous electrode into left ventricle for cardiac pacemaker |
| 38368-01 | Replacement of permanent transvenous electrode of left ventricle for cardiac pacemaker |
| 38368-03 | Replacement of transvenous electrode of left ventricle for cardiac defibrillator |
| 38390-01 | Insertion of permanent transvenous electrode into left ventricle for cardiac defibrillator |
| 38393-01 | Replacement of cardiac defibrillator generator |
| 38456-21 | Adjustment of epicardial electrode for cardiac pacemaker |
| 38456-23 | Replacement of permanent epicardial electrode for cardiac pacemaker via subxiphoid approach |
| 38456-24 | Replacement of permanent epicardial electrode for cardiac pacemaker via thoracotomy or sternotomy |
| 38456-28 | Adjustment of epicardial electrode for cardiac defibrillator |
| 38456-30 | Replacement of permanent epicardial electrode for cardiac defibrillator via subxiphoid approach |
| 38456-31 | Replacement of permanent epicardial electrode for cardiac defibrillator via thoracotomy or sternotomy |
| 38654-00 | Insertion of permanent left ventricular electrode for cardiac pacemaker via thoracotomy or sternotomy |
| 38654-01 | Replacement of permanent left ventricular electrode for cardiac pacemaker via thoracotomy or sternotomy |
| 38654-03 | Insertion of permanent left ventricular electrode for cardiac defibrillator via thoracotomy or sternotomy |
| 38654-04 | Replacement if permanent left ventricular electrode for cardiac defibrillator via thoracotomy of sternotomy |
| 90203-00 | Adjustment of transvenous electrode for cardiac pacemaker |
| 90203-02 | Adjustment of left ventricular electrode for cardiac pacemaker via thoracotomy, sternotomy or subxyphoid approach |
| 90203-05 | Adjustment of cardiac pacemaker generator |
| 90203-06 | Adjustment of cardiac defibrillator generator |
| 90203-08 | Adjustment of transvenous electrode for cardiac defibrillator |
| 90203-09 | Adjustment of left ventricular electrode for cardiac defibrillator via thoracotomy, sternotomy or subxyphoid approach |
| 90219-00 | Revision or relocation of skin pocket for cardiac pacemaker or defibrillator |

**Table S3: Antibiotic medicines**

| ATC Code | Definition |
| --- | --- |
| J01A | Tetracyclines |
| J01C | Beta-lactam antibacterials, penicillins |
| J01D | Other beta-lactam antibacterial |
| J01F | Macrolides, lincosamides and streptogramins |
| J01G | Aminoglycoside antibacterials |
| J01M | Quinolones |
| JO1X | Other antibacterials |
